# Bedinvetmab, alone or in combination with photobiomodulation and pulsed electromagnetic field therapy, on pain and quality of life in dogs with hip osteoarthritis

**DOI:** 10.1007/s11259-026-11133-3

**Published:** 2026-03-13

**Authors:** Letícia Orthey Cidral, Gilberto Serighelli-Júnior, Soraia Figueiredo de Souza, Ricardo Guilherme D’Otaviano de Castro Vilani

**Affiliations:** Department of Veterinary Medicine, Federal University of Paraná (UFPR), Curitiba, Paraná Brazil

**Keywords:** bedinvetmab, canine osteoarthritis, chronic pain, pulsed electromagnetic field therapy, photobiomodulation

## Abstract

Osteoarthritis (OA) is one of the main causes of chronic pain in dogs, significantly impairing mobility and quality of life. This study aimed to evaluate the analgesic efficacy of bedinvetmab, administered alone and in combination with physiotherapy using photobiomodulation (PBM) and pulsed electromagnetic field therapy (PEMF), in dogs with hip OA. This was a prospective, randomized, comparative effectiveness trial involving 30 dogs allocated into two groups: BPG (bedinvetmab + physiotherapy) and BG (bedinvetmab). In the BPG, physiotherapy sessions were performed twice a week throughout the 90-day protocol. Pain was assessed through algometry and the Canine Brief Pain Inventory (CBPI) completed by the owners. Both groups showed significant clinical improvement (*p* < 0.001). In the CBPI, both the Pain Severity Score (PSS) and Pain Interference Score (PIS) decreased significantly from baseline to day 90 in both groups. In algometry, the BPG showed a significant increase in pain threshold as early as day 15 (D15) and was superior to the BG from day 30 (D30; *p* = 0.027), with more pronounced differences at days 60, 75, and 90 (D60, D75, and D90; *p* < 0.001). These findings provide preliminary evidence that the combination of bedinvetmab and physiotherapy enhances pain control and promotes better quality of life in dogs with OA.

**Supplementary Information:**

The online version contains supplementary material available at 10.1007/s11259-026-11133-3.

## Introduction

Osteoarthritis (OA) represents one of the leading causes of chronic pain in dogs, particularly in elderly animals (Neagu et al. 2023). It is a progressive and degenerative condition characterized by articular cartilage degradation, inflammatory responses and chronic pain, leading to biomechanical limitation and progressive loss of function (Szponder et al. 2022). OA can be classified as primary, usually idiopathic and associated with age and obesity, or as secondary, the most common form, resulting from joint injuries and dysplasias. Genetic predisposition, inadequate diet and sedentary lifestyle may act as contributing factors (Anderson et al. 2020).

In the United Kingdom, an annual prevalence of 2.5% has been reported, equating to around 200.000 cases per year. Moreover, the condition affects approximately 11.4% of the lifespan of affected dogs, with direct repercussions on their quality of life (Anderson et al. 2018). It is noteworthy that recent prevalence estimates may be underestimated due to limitations in reporting methods. These include dogs that did not receive veterinary care, absence of a formal OA diagnosis, and incomplete data recording by veterinarians (Anderson et al. 2020). In a cohort of 500 dogs screened using an owner‑reported osteoarthritis checklist, 38% were clinically confirmed to have OA, indicating that a substantial number of cases might otherwise remain undetected and unreported in routine practice (Wright et al. 2022). Chronic pain invariably interferes with mobility, posture, quality of life, sleep, and social interactions (Lascelles et al. 2019). Nonetheless, it is often underestimated and regarded as a natural consequence of aging (Monteiro et al. 2023). Early signs of OA in dogs are generally subtle and progressive, reinforcing the importance of regular pain assessment by veterinarians and the involvement of caregivers in detecting behavioral changes at home (Belshaw et al. 2020).

From the owner’s perspective, treatment is considered effective when it is readily available, easy to administer, fits into daily routines, and requires less frequent dosing. When these criteria are not met, caregivers may avoid or discontinue treatment, underscoring the importance of effective and practical pain management strategies (Gildea et al. 2024). In contrast, inadequate management may lead to systemic complications, caregiver emotional burden, and, consequently, euthanasia (Spitznagel et al. 2022), which is common in cases of chronic pain and degenerative diseases (Mota Rojas et al. 2023). Collaborative follow-up between veterinarians and caregivers facilitates the adaptation of therapeutic strategies, slowing disease progression and improving quality of life (Belshaw et al. 2020).

Over the last decade, several advances have expanded the pharmacological treatment options available for dogs with OA, including non-steroidal anti-inflammatory drugs (NSAIDs), piprants, monoclonal antibodies, adjunctive analgesics, structure-modifying OA drugs, and regenerative therapies (Pye et al. 2022). NSAIDs remain widely used as a standard treatment for pain management in dogs with OA. However, despite their well-established efficacy, long-term administration is associated with clinically relevant adverse effects, and analgesic response is often insufficient when these drugs are used as monotherapy (Lascelles et al. 2005; Enomoto et al. 2019; Corral et al. 2021).

Among available therapeutic options, the caninized monoclonal antibody (mAb) bedinvetmab has emerged as an important approach for the management of osteoarthritis-related pain in dogs (Enomoto et al. 2019; Lascelles et al. 2019; Reid et al. 2024; della Rocca et al. 2025) Bedinvetmab acts by inhibiting nerve growth factor (NGF), thereby interrupting NGF/TrkA signaling involved in the development and maintenance of chronic pain (Enomoto et al. 2019; Lascelles et al. 2019). Clinical studies have demonstrated significant analgesic efficacy and functional improvement in dogs with OA treated with bedinvetmab (Corral et al. 2021; Michels et al. 2023), with a favorable safety profile when compared with traditional NSAIDs (Enomoto et al. 2019).

Given the complexity of chronic pain pathophysiology, OA management should be based on a multimodal approach that integrates pharmacological and non-pharmacological strategies, aiming at pain reduction, preservation of joint function, and improvement of quality of life. Current guidelines and reviews consistently emphasize that effective OA management requires combining analgesia with weight control, nutritional strategies, and structured physical rehabilitation (Gruen et al. 2022; Mosley et al. 2022; Cachon et al. 2023). Core components of multimodal care include body weight management, supplementation with fatty acids such as omega-3, adapted physical exercise, and physiotherapeutic modalities. These modalities encompass therapeutic exercise, hydrotherapy, photobiomodulation therapy, and electromagnetic field therapy, which are increasingly incorporated into comprehensive OA treatment plans (Mille et al. 2023; Monteiro et al. 2023; Pye et al. 2024).

Among physiotherapeutic modalities, photobiomodulation therapy (PBM), when applied to arthritic joints, has been reported to reduce pro-inflammatory mediators, improve tissue oxygenation, and stimulate mitochondrial activity, which may contribute to tissue repair and analgesia (Hamblin, 2019). PBM may also decrease inflammatory cells in synovial fluid and modulate bradykinin receptors, attenuating nociceptive sensitivity (Bortone et al. 2008). In canine OA, PBM has been investigated as an adjunctive modality, with clinical studies reporting reductions in pain and improvements in mobility and functional outcomes (Looney et al. 2018; Alves et al. 2022; Barale et al. 2022). In addition, evidence from in vitro and in vivo studies suggests that PBM may modulate inflammatory pathways and cellular processes involved in OA, reinforcing its biological plausibility in musculoskeletal disorders, although clinical effects remain protocol-dependent and require further investigation (Kunimatsu et al. 2025).

Pulsed electromagnetic field therapy (PEMF) has been explored as a non-pharmacological modality capable of influencing bioelectrical and cellular processes. Experimental and translational studies indicate that PEMF may modulate inflammatory signaling pathways and cartilage metabolism, although the underlying mechanisms remain incompletely understood (Yang et al. 2020). At the cellular level, experimental models suggest that PEMF may influence chondrocyte activity and extracellular matrix metabolism, contributing to its proposed disease-modifying potential (Yang et al. 2020). Analgesic effects associated with PEMF have been reported in experimental and clinical contexts and are hypothesized to involve modulation of nociceptive signaling, potentially through effects on ion channel activity and neuronal excitability (Rajalekshmi and Agrawal 2024). In veterinary medicine, PEMF has been investigated as a standalone intervention in dogs with hip OA, with preliminary clinical studies describing pain-related improvements following treatment (Kühl et al. 2025; Šutalo et al. 2025). However, available evidence remains limited, and direct mechanistic data in canine OA are scarce. Systematic reviews and clinical studies in human OA have also reported reductions in pain and functional improvement following PEMF therapy, supporting its potential relevance as an adjunctive modality, although protocols and outcomes remain heterogeneous (Cianni et al. 2024).

To the authors knowledge, there are no studies associating PBM and PEMF therapies in dogs with hip OA. Thus, while PBM exerts direct photobiological effects on metabolic and inflammatory processes, PEMF induces electromagnetic alterations capable of modulating the inflammatory and structural response of cartilage. Considering that bedinvetmab blocks NGF/TrkA signaling and interrupts pain transmission (Enomoto et al. 2019), the combination of these modalities provides distinct and complementary mechanisms: blockade of the nociceptive pathway, modulation of inflammation, and stimulation of tissue repair.

In this context, the present study aims to evaluate whether the association of PBM and PEMF enhances the analgesic effects of bedinvetmab and contributes to improved quality of life in dogs with OA compared with the monoclonal antibody alone.

## Materials and methods

This study was designed as a prospective, randomized, open-label experimental clinical trial. Objective pain assessment was performed by the evaluator using algometry, while subjective pain assessment was obtained through owner-completed CBPI questionnaires (Brown et al. 2013).

The project was approved by the Ethics Committee on the Use of Animals (CEUA) of the Federal University of Paraná, in accordance with Law No. 11.794 of 10/08/2008. The study was conducted on domiciled dogs in the city of Curitiba, Paraná, Brazil.

**Fig. 1:**
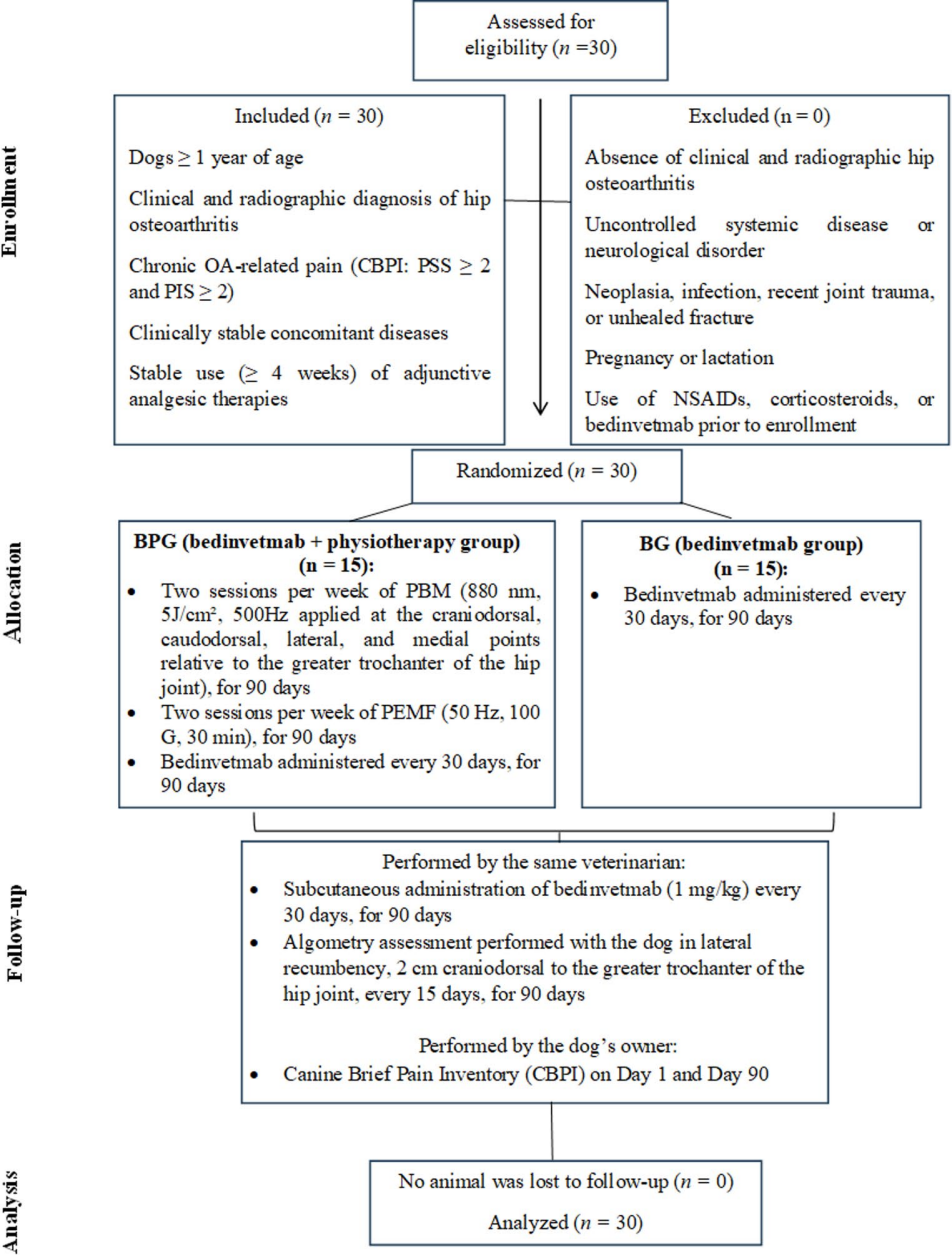
CONSORT-type flow diagram showing the eligibility criteria, inclusion (*n* = 30), absence of exclusions, random allocation into two groups (BPG: bedinvetmab + physiotherapy, *n* = 15; BG: bedinvetmab, *n* = 15), treatment protocols, clinical follow-up (algometry and CBPI questionnaire), and final analysis of participants (*n* = 30). Source: authors’ own work (2025)

Thirty dogs with a clinical and radiographic diagnosis of hip OA were enrolled in the study (*n* = 30). Dogs were randomly allocated into two groups: a bedinvetmab-only group (BG; *n* = 15) and a bedinvetmab plus physiotherapy group (BPG; *n* = 15), which additionally received PBM and PEMF twice weekly (Fig. 1). Both treatments were carried out over 90 days. The owners were not informed about the existence of different experimental groups or about the allocation of their dogs to a specific treatment group.

The inclusion criteria comprised dogs with chronic pain related to OA, with clinical and radiographic diagnosis, that presented a score equal to or greater than two in both the pain severity and functional interference domains, according to CBPI evaluation. In addition, a minimum age of one year, regardless of sex, breed, or weight, was required. Dogs with clinically stable concomitant diseases (early-stage cardiac or endocrine disorders under ongoing treatment) were eligible for inclusion, provided that routine screening tests (complete blood count and urinalysis) were within reference ranges. This criterion aimed to ensure systemic stability at enrolment and to reduce confounding from uncontrolled systemic illness, rather than to exclude the presence of comorbidities.

Dogs that were already receiving pregabalin, gabapentin, or nutraceuticals such as omega-3 fatty acids and type II collagen were also eligible for inclusion. However, these treatments had to be maintained at stable doses and frequencies for at least four weeks prior to enrollment. This requirement was intended to ensure that any therapeutic effects from these adjuvant treatments were already established and would not interfere with the evaluation of the experimental protocol during the study. No washout period was required for these therapies, and no changes to these medications were allowed throughout the study period.

Dogs without clinical and radiographic diagnosis of OA in the hip joint, receiving uncontrolled concomitant treatments, pregnant or lactating females, breeding animals, with lameness associated with neoplasia, degenerative myelopathy, immunological disorders, infections, recent joint trauma, unhealed fractures were excluded. Dogs receiving corticosteroids or NSAIDs within 14 days prior to study enrollment were also excluded. This washout period was adopted to avoid potential interference of residual anti‑inflammatory or analgesic effects with the evaluation of the primary treatment modalities. Patients who had previously received bedinvetmab were likewise excluded.

Bedinvetmab (Librela^®^, Zoetis) was provided as a ready-to-use commercial formulation in a 1 mL single-dose vial, preservative-free, stored under refrigeration between 2 °C and 8 °C. The dosage was adjusted to each patient’s body weight, ensuring administration of 0.5 to 1.0 mg/kg, according to the manufacturer’s recommendations. In both groups, the mAb was administered subcutaneously on days (D): D1, D30, D60, and D90.

### Clinical and functional assessments

On the first day of treatment (D1), all dogs underwent clinical and orthopedic evaluation (including measurement of vital parameters, inspection, palpation, joint manipulation), hip radiographs, neurological examination, gait analysis, and weight-bearing assessment. Based on the radiographs, hip dysplasia was classified according to the Federation Cynologique Internationale (FCI) grading system, ranging from grade A (no signs of dysplasia) to grade E (severe dysplasia) (Supplementary File 1).

In addition to hip joint evaluation, a general orthopedic screening and complementary radiographic exams were performed to identify signs of OA in other joints, which were recorded along with data such as age, sex and breed. The presence of myofascial spinal pain was also assessed during the clinical examination through myofascial palpation and the application of digital pressure over spinous and articular processes, allowing differentiation between axial-origin pain and pain related to facet joints. These findings were documented for descriptive analysis.

However, only dogs with clinically and radiographically confirmed hip OA were included, and pain assessments focused specifically on the hip region. The owners received guidance on environmental and nutritional management and daily 10-minute walks, as well as instructions to report adverse effects. Adverse events were monitored throughout the study, including changes in body temperature, pruritus, and mental state.

Algometry was performed every 15 days (D1, D15, D30, D45, D60, D75, and D90) in both groups, using a Topcat^®^ pressure algometer with a 3 mm probe and maximum force of 25 N. Evaluations were conducted in a calm environment, with the dogs positioned comfortably in lateral recumbency and preferably at rest. The stimulus was applied 2 cm craniodorsal to the greater trochanter of the hip joint, on the side most affected by OA (clinically defined as the one with the greatest pain or discomfort). Pressure was increased progressively and continuously at an approximate rate of 1 N/second, resulting in an average duration of 3 to 5 s per measurement, until a behavioral response was observed. The procedure followed the luminous indications of the device and the manufacturer’s guidelines. To ensure reproducibility, the measurement was performed only once per session, without consecutive repetitions, in order to avoid peripheral sensitization or nociceptive fatigue. The response to the stimulus was considered positive when the animal showed behaviors such as limb withdrawal, vocalization, or head orientation toward the stimulated point (Knazovicky et al. 2016).

In the BP, physiotherapy consisted of two modalities: PBM and PEMF. PBM was delivered using a diode laser device (Globus^®^, LaserVet 1000) with a wavelength of 808 nm, operated in pulsed mode (500 Hz) and adjusted to a power output of 1 W, consistent with previously published protocols using the same device for canine osteoarthritis (Barale et al. 2022). The laser was applied in point mode at four standardized locations surrounding the hip joint (craniodorsal, caudodorsal, lateral, and medial to the greater trochanter). Each point received an energy density of 5 J/cm², assuming a spot size of approximately 1 cm², resulting in an average exposure time of 5 s per point. PBM was applied directly to the skin following standard safety procedures, including the use of protective eyewear by the operator and avoidance of direct ocular exposure in the animals. These parameters were selected to ensure a non-thermal, biologically effective photobiomodulatory dose aimed at modulating inflammation and promoting tissue repair in musculoskeletal conditions.

PEMF was applied immediately after PBM using the Globus^®^ 4000 device, designed for veterinary use. Two pairs of solenoids were positioned dorsally over the hip joints, aligned with the anatomical region of greatest joint involvement. The electromagnetic field was delivered at a frequency of 50 Hz and intensity of 100 Gauss, for 30 min per session. The selected frequency is consistent with previous studies investigating the analgesic effects of PEMF in dogs with hip OA (Kühl et al. 2025). The application protocol followed the manufacturer’s instructions and standard recommendations for musculoskeletal rehabilitation using PEMF (Yang et al. 2020; Cianni et al. 2024).

During each session, dogs were comfortably positioned in lateral recumbency in a quiet environment to reduce stress and movement. The solenoids were securely fastened to ensure adequate field targeting while allowing patient comfort. Both PEMF and PBM were administered twice weekly over the 90-day treatment period, totaling 24 sessions.

## Pain and life quality evaluation

Chronic pain was evaluated by the owners using the CBPI, which includes three domains: PSS, PIS, and a single-item overall Quality of Life (QoL) assessment. The PSS consists of four items scored from 0 to 10, and the PIS consists of six items scored from 0 to 10. For each domain, scores were calculated as the arithmetic mean of the corresponding items, in accordance with the original validation and recommended use of the instrument. Higher mean scores indicate greater pain severity or interference with daily activities. The QoL item was analyzed separately as an ordinal variable. The CBPI was administered at baseline (D1) and at the end of the study (D90), preferably by the same owner.

### Statistical analysis

For the analysis of algometric data between D1 and D90, a linear mixed model with random intercept per animal was used, suitable for handling repeated measures and controlling inter-individual variability. The fixed variables were time and experimental group. Degrees of freedom and p-values were obtained by the Satterthwaite method, which provides greater accuracy in statistical inference, especially in small samples and complex data structures. p-values below 0.05 were considered statistically significant. Data distribution was assessed for normality, and given the non-normal distribution of algometric data, baseline comparisons between groups were performed using nonparametric tests.

In addition, to assess whether the experimental groups were balanced in terms of the radiographic severity of hip dysplasia, a chi-square test was conducted using the FCI classification (grades C, D, and E). To evaluate baseline balance in the use of adjuvant medications with potential analgesic effects (such as pregabalin, gabapentin, omega-3 supplementation, and type II collagen), Fisher’s exact test was applied due to the small sample size in some categories.

The adequacy of the linear mixed model was assessed using the Akaike Information Criterion (AIC) and the Bayesian Information Criterion (BIC). The full model, including time, group, and their interaction, was compared with reduced models in which one or more terms were removed. Model selection favored the full model, which showed superior AIC performance and better captured the experimental variability.

To assess statistical power, a post hoc power analysis was performed based on D90 data for each primary outcome. Using R software (version 4.4.1) and the t-test for two independent samples (α = 0.05, *n* = 15 per group), effect size was estimated by Cohen’s d (0.2 small, 0.5 moderate, 0.8 large). Power values above 80% were considered adequate.

Data from the CBPI questionnaire were analyzed separately by domain. PSS and PIS, calculated as mean domain scores, were treated as continuous variables and described using median, minimum and maximum. Within-group comparisons between D1 and D90 were performed using the paired Wilcoxon test. The QoL item, analyzed as an ordinal variable, was evaluated descriptively and compared over time using nonparametric methods. The proportion of dogs presenting clinically relevant pain, defined as mean PSS ≥ 2 and PIS ≥ 2, was described at baseline and at D90. All analyses were performed using Python (v3.10) and R (v4.4.1).

## Results

During the study, no animal exhibited adverse reactions related to bedinvetmab. At every visit, dogs were evaluated for signs of musculoskeletal alterations (sudden lameness, joint instability, fractures, polyarthritis), neurological changes (proprioceptive deficits, altered reflexes, tremors), gastrointestinal disturbances (vomiting, diarrhea, hyporexia), and dermatological manifestations (pruritus, erythema, alopecia). All dogs maintained good tolerance to the treatment throughout the experimental protocol.

A total of 30 dogs were included in the study, comprising 14 males (46.6%) and 16 females (53.3%), with ages ranging from 3 to 19 years. Baseline age and body weight characteristics of both groups are summarized in Table 1. In the BPG, there were 7 males and 8 females, whereas in the BG there were 8 males and 7 females. No significant difference in sex distribution was observed between groups (chi-square test, *p* = 1.0).

Age distribution analysis showed a slightly higher proportion of dogs aged 14 years or older in the BPG (*n* = 6) compared with the BG (*n* = 3). In the 8–13 year age range, the BG included more dogs (*n* = 8) than the BPG (*n* = 5), while both groups had the same number of dogs younger than eight years (*n* = 4). These differences were not statistically significant (chi-square test, *p* = 0.429).

Regarding baseline radiographic severity of hip dysplasia, according to the FCI system, only grades C, D, and E were observed, with no cases of grades A or B. The distribution of dysplasia severity did not differ significantly between groups (chi-square test, *p* = 0.894), indicating balanced baseline OA burden (Supplementary Table 1).

All dogs enrolled in the study presented hip joint OA confirmed by radiographic and clinical examination. Many cases were also associated with involvement of additional joints, including pelvic limb (PL) joints, such as femorotibiopatellar and tarsal, thoracic limb (TL) joints, such as glenohumeral and humeroradioulnar, and myofascial spinal pain.

A total of 13 dogs (86.7%) in the BPG and 12 dogs (80%) in the BG met the criteria for multiple joint osteoarthritis (MJOA), defined as the involvement of three or more distinct joint sites (Nelson et al. 2025). Myofascial spinal pain was observed in 66.6% of BPG dogs and 60% of BG dogs. Considering the entire study population (*n* = 30), 15 dogs exhibited simultaneous pain in TL, PL, and spine, while four dogs presented only spinal myofascial pain and pelvic limb involvement. No animal showed pain in TL and PL without also manifesting spinal myofascial pain.

Regarding body size, BPG included six large dogs (40%), five medium-sized dogs (33.3%), and four small dogs (26.6%). In BG, six dogs were large (40%), two medium (13.3%), and seven small (46.6%). The sample included purebred dogs such as Shih-Tzu (*n* = 7), Golden Retriever (*n* = 3), Labrador Retriever (*n* = 2), German Shepherd (*n* = 2) and Yorkshire Terrier (*n* = 2), among others, as well as mixed-breed dogs (*n* = 9), which represented 30% of the total sample. All dogs in the BPG were neutered, whereas in the BG, two animals (13.3%) were intact.

**Table 1:** Comparison of age and weight between study groups in osteoarthritic dogs treated with bedinvetmab associated (BPG) or not (BG) to physiotherapy. Mean and standard deviation of age and weight in dogs from the BPG and BG groups, and comparision between groups using Student’s t-test

	Age (Years)	Weight (Kg)
BPG	11.5 ± 4.9	17.7 ± 9.9
BG	9.9 ± 3.9	13.7 ± 10.2
P value	0.350	0.278

Data expressed as mean ± standard deviation. *BPG* bedinvetmab + physiotherapy; *BG* bedinvetmab. No significant differences were found between groups (Student's t-test; *p* > 0.05)

During the study period, participating dogs were allowed to continue previously prescribed adjuvant medications for chronic pain management, including anticonvulsant agents (pregabalin and gabapentin). Eight animals were receiving pregabalin administered every 24 h, five in the BG and three in the BPG. Gabapentin was used by two dogs, administered every 12 h, with equal distribution between groups (one in each).

The use of dietary supplements was also permitted, including omega-3, administered every 24 h to seven dogs (three in BG and four in BPG), and type II collagen, used by six dogs (four in BG and two in BPG).

Baseline distribution of these concomitant therapies between groups was evaluated using Fisher’s exact test, which revealed no statistically significant differences for pregabalin (*p* = 0.681), gabapentin (*p* = 1.000), omega-3 (*p* = 1.000) and type II collagen (*p* = 0.651).

Pain-related outcomes are presented below, with algometry as the primary endpoint. Table 2 presents the pain threshold measured by algometry in the BPG and BG at seven different time points (D1 to D90).

**Table 2:** Algometric comparison between combined therapy with physiotherapy and bedinvetmab (BPG) and isolated bedinvetmab (BG) treatment in osteoarthritic dogs over time (D1–D90)

Group	D1	D15	D30	D45	D60	D75	D90
BPG	4.0 [1.8–4.7]	4.9 [2.2–7.3]	5.0 [2.5-8.0]	5.3 [3.0-6.6]	5.6 [3.0-7.5]	5.9 [3.4–7.8]	6.0 [3.6–7.9]
BG	3.4 [1.5–5.5]	4.4 [2.5–6.8]	3.8 [2.1–6.1]	4.0 [2.3-7.0]	4.2 [2.2–6.2]	4.3 [2.9–6.9]	4.2 [3.0-6.9]
*p* value	*p* = 0.954	*p* = 0.595	*p* = 0.027	*p* = 0.047	*p* < 0.001*	*p* < 0.001*	*p* < 0.001*

*Median*, minimum and maximum values of pressure algometry thresholds (Newtons) for each group (BG and BPG) from D1 to D90

**Fig. 2:**
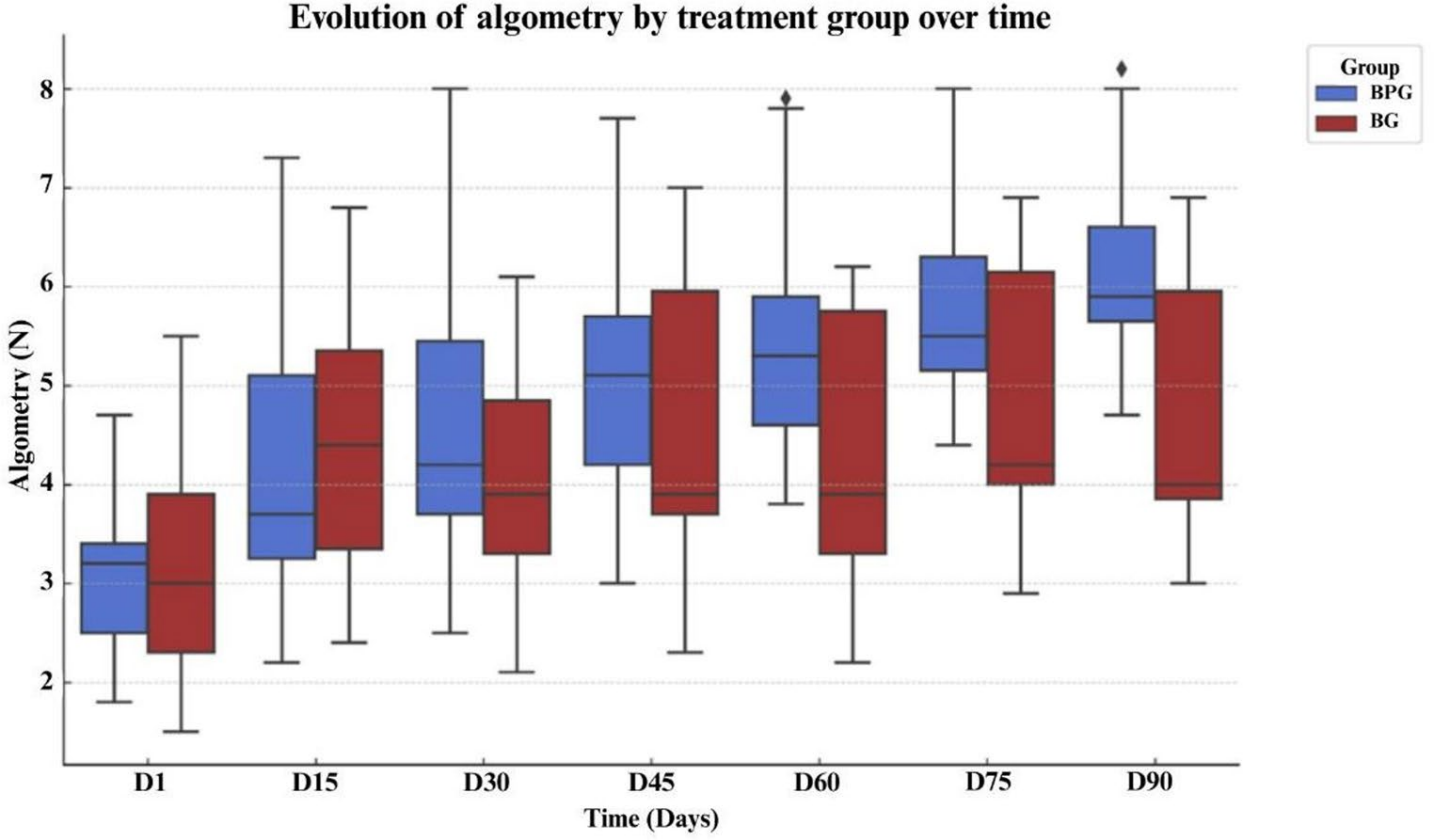
Distribution of algometry values (N) in dogs with hip osteoarthritis treated with bedinvetmab (BG, *n* = 15) or bedinvetmab combined with physiotherapy using photobiomodulation and pulsed electromagnetic field therapy (BPG, *n* = 15), evaluated at D1, D15, D30, D45, D60, D75, and D90. The boxes represent the interquartile range (Q1–Q3), the horizontal line indicates the median, the vertical whiskers show the minimum and maximum values, and the isolated points represent outliers. Source: authors’ own data, 2025.

The linear mixed model with a random intercept per animal, using the Satterthwaite method, revealed significant effects of time and group–time interaction on pain thresholds assessed by algometry (Fig. 2). In the BPG, a significant increase in the median algometry values was observed from D15 onward, with a statistical difference compared to D1 (*p* < 0.001), demonstrating progressive improvement. The comparison between groups showed that at D1 there was no significant difference (*p* = 0.954), indicating initial equivalence. However, the BPG exhibited significant differences compared to the BG starting at D30 (*p* = 0.027), with more pronounced results at D60 (*p* < 0.001), D75 (*p* < 0.001), and D90 (*p* < 0.001).

Table 3 presents the mean per item scores of the PSS, PIS, and QoL domain of the CBPI, completed by the owners at baseline (D1) and at the end of the study (D90). A statistically significant reduction over time was observed in both groups for PSS and PIS (*p* < 0.0001).

**Table 3:** Comparison of Canine Brief Pain Inventory (CBPI) domain scores (mean per item) in dogs treated with the bedinvetmab alone (BG) or bedinvetmab combined with physiotherapy (BPG) over time (D1 to D90)

Variable	Time	BG Median (min-max)	BPG Median (min-max)	*p* value
PIS (0–10)	D1	8.67 [5.83–9.83]	7.33 [0.67–10.00]	0.197
	D90	1.83 [0.00–3.50]	2.00 [0.50–6.50]	0.309
PSS (0–10)	D1	5.33 [4.67–6.50]	4.83 [4.00–6.50]	0.096
	D90	1.17 [0.83–2.50]	2.00 [0.17–10.00]	0.546
QoL (1–5)	D1	1 [1–2]	1 [1–2]	0.036*
	D90	4 [3–5]	5 [3–5]	0.007*

*PIS* pain interference score (mean of 6 items, 0–10); *PSS *pain severity score (mean of 4 items; 0–10); *QoL *quality of life (ordinal score, 1–5)

At baseline (D1), all dogs met the inclusion criteria for clinically relevant chronic pain, defined as mean PSS ≥ 2 and mean PIS ≥ 2. By D90, this proportion had decreased markedly in both groups, with only one dog per group (7%) still meeting these criteria.

**Fig. 3:**
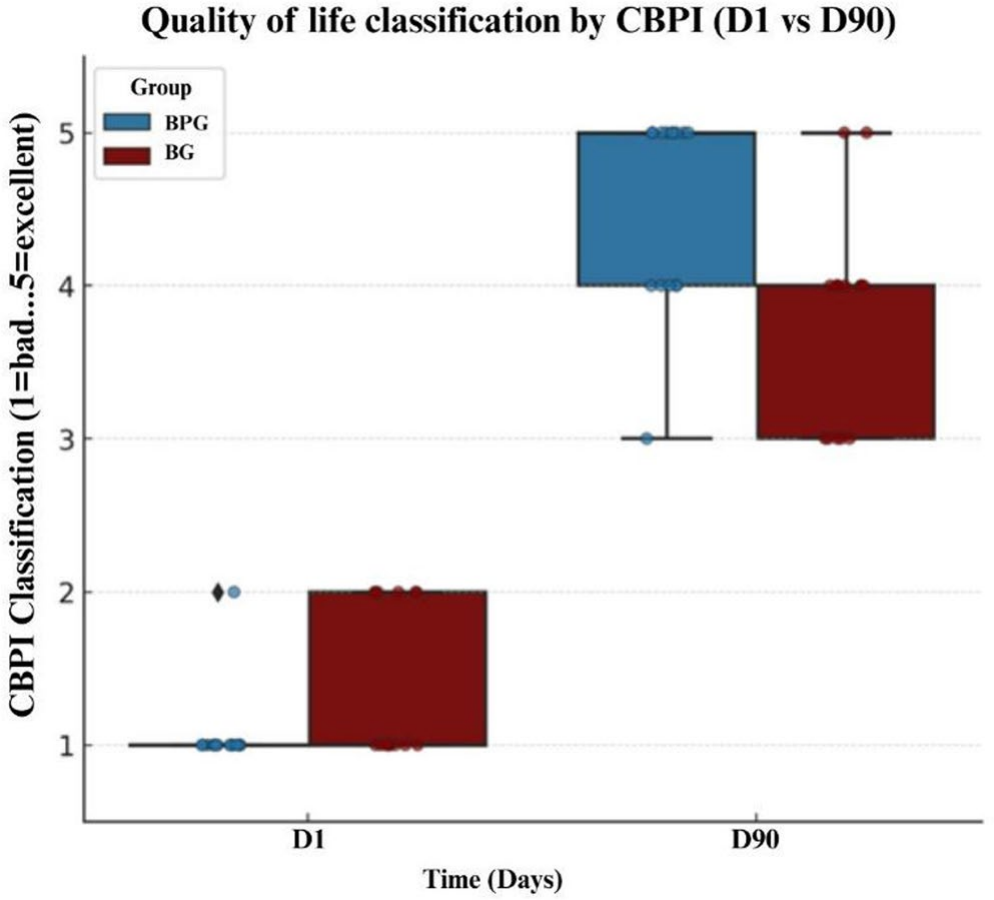
Distribution of the quality of life classification assigned by the owners according to the Canine Brief Pain Inventory (CBPI; 1 = poor, 2 = fair, 3 = good, 4 = very good, 5 = excellent) in dogs with hip osteoarthritis treated with bedinvetmab (BG, *n* = 15) or bedinvetmab combined with physiotherapy using photobiomodulation and pulsed electromagnetic field therapy (BPG, *n* = 15). Evaluations were performed at the beginning of the study (D1) and after 90 days (D90). The boxes represent the interquartile range (Q1–Q3), the horizontal line indicates the median, the vertical whiskers show the minimum and maximum values, and the isolated points correspond to outliers. Source: authors’ own data, 2025.

Regarding quality of life assessment (Fig. 3), owner-reported QoL improved substantially in both groups. At D90, 60% of dogs in the BPG were classified as “excellent” and 33% as “very good,” whereas in the BG, 13% were rated as “excellent,” 53% as “very good,” and 33% as “good.” At baseline, most dogs in both groups were classified as having “poor” or “fair” quality of life. Conversely, in the BG group, only 13% reached the “excellent” classification, while the remaining were rated as “very good” (53%) and “good” (33%). At D1, 60% of the BG dogs were classified as “poor” and 40% as “fair.”

The complete linear mixed model showed superior performance, with AIC = 456.28 and BIC = 506.48. The post hoc power analysis demonstrated a statistical power of 93.4% (*d* = 1.31) for the pain threshold outcome assessed by algometry and 42% (*d* = 0.66) for the pain score perceived through the CBPI, considering α = 0.05 and *n* = 15 per group.

## Discussion

Chronic pain management with the anti-NGF mAb, either alone or combined with PBM and PEMF, was associated with statistically significant changes in pain thresholds over three months in dogs with hip OA. The present study suggests a potential cumulative analgesic effect of adding PBM and PEMF to bedinvetmab within a multimodal treatment approach, particularly in dogs presenting with higher baseline pain scores.

Regarding safety, although recent studies have reported musculoskeletal and neurological adverse events in dogs treated with bedinvetmab, no adverse events were observed in the animals evaluated during the three-month follow-up of the present study. However, the limited sample size and short duration of follow-up preclude any conclusions regarding the safety profile or incidence of adverse events. Reports from pharmacovigilance databases and post-marketing surveillance have described musculoskeletal and neurological events associated with bedinvetmab use (FDA 2024; Farrell et al. 2025), which should be interpreted in the context of increasing clinical exposure and active reporting systems. Therefore, caution remains warranted when using bedinvetmab, particularly in dogs with comorbidities, and larger, longer-term studies are required to adequately assess safety outcomes.

No patient presented simultaneous pain in forelimbs and hindlimbs without also manifesting myofascial spinal pain, indicating its frequent role as a compensatory overload site in hip OA. This pattern reflects weight redistribution and biomechanical adaptations that affect secondary joints such as stifles and tarsi (Marcellin-Little et al. 2025). The early onset of trigger points in the quadriceps femoris and paravertebral muscles suggests these are the first to respond to overload (Formenton et al. 2025). Additionally, even with preserved ground reaction forces in the affected limb, the contralateral limb tended to bear greater load, evidencing asymmetric compensations that impair gait and highlight the importance of integrative and systemic therapeutic approaches (Alves et al. 2022).

From a methodological standpoint, baseline algometric values were comparable between groups, indicating initial sample homogeneity and reducing the likelihood of allocation bias. The choice of the full linear mixed model was based on the lowest AIC value (456.28), which prioritizes data fit with less penalization for complexity. Although the BIC (506.48) favored a reduced model, the full model was retained due to its greater ability to capture time–group interactions, which were fundamental to the study objectives. At baseline (D1), pain thresholds assessed by algometry were similar between groups (BPG: 4.0 [1.8–4.7] N; BG: 3.4 [1.5–5.5] N; *p* = 0.954), as shown in Table 2.

Within this context, the present study suggests a potential additional benefit of combining PBM and PEMF with bedinvetmab in the management of chronic pain associated with OA. Based on the linear mixed model, a statistically significant group × time interaction was detected, indicating that changes in pain thresholds over time differed between treatment groups. Between-group comparisons showed no significant difference at baseline (D1; *p* = 0.954), confirming initial homogeneity. From day 30 onward, dogs in the BPG group exhibited higher pain thresholds compared with the BG group (D30: *p* = 0.027; D45: *p* = 0.047), with more pronounced between-group differences observed at days 60, 75, and 90 (all *p* < 0.001), as presented in Table 2. Taken together, these findings indicate an association between the addition of PBM and PEMF and greater modulation of pain thresholds over time, while acknowledging that methodological limitations preclude definitive causal conclusions.

With respect to the temporal pattern of response, at days 30, 60, and 90, corresponding to the reapplication of bedinvetmab, a slight but statistically non-significant reduction in pain thresholds assessed by algometry was observed. According to official prescribing information, bedinvetmab shows a terminal elimination half-life of approximately two weeks in dogs treated at the licensed dose. While this pharmacokinetic profile may contribute to temporal variability in clinical response, no pharmacokinetic data were collected in the present study, and this interpretation remains speculative. Importantly, analgesic responses remained stable over time, with no evidence of clinically relevant loss of efficacy.

The biological plausibility of these findings is supported by experimental and clinical evidence on the effects of PBM and PEMF. PBM, both in vivo and in vitro, has been shown to stimulate connective tissue cells such as osteoblasts, chondrocytes, and tenocytes, promoting proliferation, cell migration, and gene expression favorable to regeneration. Repeated protocols enhance therapeutic response by stimulating collagen synthesis, activation of growth factors, increased microcirculation, and angiogenesis (Shaikh Kader and Houreld 2022). Consistently, a randomized double-blinded clinical trial in dogs with hip OA demonstrated significant pain reduction and functional improvement as early as day 8, with effects maintained and progressively intensified up to day 90 (Alves et al. 2024).

In the present study, the progressive improvement in pain tolerance observed in dogs receiving PBM and PEMF was associated with repeated stimulation over time, suggesting a potential cumulative effect on neuromuscular adaptation and pain modulation. Evidence from human medicine supports the biological plausibility of these mechanisms. A systematic review and meta-analysis by Yang et al. (2020) demonstrated that PEMF reduces pain and stiffness, improves physical function, and modulates inflammatory cytokines and extracellular matrix metabolism in patients with OA. However, direct extrapolation from human studies to canine models is limited, and such evidence should be interpreted as supportive of potential mechanisms rather than as direct proof of comparable clinical effects in dogs.

Beyond peripheral analgesic effects, the combination of PBM and PEMF may also act on endogenous central inhibitory mechanisms, similar to conditioned pain modulation (CPM). In simplified terms, this means that a painful stimulus can activate descending pathways that reduce pain perception in other body regions through neurotransmitter release such as serotonin and endorphins (Yarnitsky 2015). PBM further contributes by increasing ATP production, nitric oxide release, and modulation of reactive oxygen species, resulting in anti-inflammatory effects and reduced peripheral and central nociceptive excitability (Chow et al. 2021). This combination of physiological effects may explain the superior clinical stability and better quality-of-life perception observed in the BPG, corroborating international WSAVA recommendations for multimodal chronic pain management (Monteiro et al. 2023).

Human clinical evidence further supports this rationale. In a systematic review of 17 randomized clinical trials involving 1.197 OA patients, PEMF reduced pain by an average of 60% (VAS scale) and improved function by 42% (WOMAC scale), regardless of protocol variations, in addition to reducing NSAID requirements and improving quality of life (Cianni et al. 2024).

Taken together, the present findings suggest that monotherapy may be limited in the management of chronic OA pain, as it does not fully address the multifactorial nature of the disease (Gruen et al. 2022). The addition of PBM and PEMF to bedinvetmab was associated with improved pain-related and functional outcomes in the present study, providing preliminary indication of the potential value of integrated multimodal strategies for canine OA management. Similar improvements have been reported in dogs with OA treated with combinations of pharmacological, nutritional, and rehabilitative interventions (Enomoto et al. 2024), supporting a multimodal framework that integrates analgesia, nutrition, weight management, and rehabilitation (Gruen et al. 2022).

A practical framework for multimodal treatment should therefore be adapted to the stage of disease progression, emphasizing early functional rehabilitation, appropriate monitoring tools, and individualized therapy (Marcellin-Little et al. 2025). Such an approach reinforces the role of physiotherapy in the context of canine chronic pain and long-term quality of life.

As with algometry, the results obtained through the CBPI questionnaire should be interpreted with caution. Although CBPI is a validated instrument for assessing chronic pain in dogs, it is inherently subjective and highly dependent on caregiver perception. In the present study, improvements in CBPI scores were observed in both treatment groups *(p* < 0.001), indicating an association with perceived pain relief over time. Previous studies have reported significant improvements in CBPI scores in dogs treated with bedinvetmab compared with placebo (Michels et al. 2023). In the present study, more pronounced improvements were reported in the group receiving physiotherapy; however, the study followed an open-label design, and although owners were not informed about the existence of different treatment groups, the absence of formal blinding may have introduced expectancy bias, particularly given the increased frequency of clinical visits in the physiotherapy group. Therefore, CBPI findings should be interpreted as associative rather than causal.

In line with these observations, improvements in owner-perceived pain and quality of life were observed in both treatment groups, as reflected by reductions in the PSS and PIS, as well as by higher QoL ratings at D90. Dogs receiving the combined PBM and PEMF protocol showed a greater concentration of lower PSS and PIS values and higher quality-of-life classifications at the end of treatment, indicating not only improved pain control but also reduced variability in owner-reported outcomes.

Pain assessments in this study, algometry and the CBPI questionnaire, while complementary, may yield different outcomes. Algometry is a quantitative, standardized tool capable of detecting changes in nociceptive threshold to mechanical stimuli. However, it is not fully objective, as it depends on the animal’s behavioral response to the stimulus (limb withdrawal or vocalization), which may introduce variability. By contrast, CBPI evaluation reflects caregiver perception of functional, emotional, and behavioral aspects not captured by algometry, and is equally relevant for understanding chronic pain.

Post-hoc power analysis reinforces this distinction between assessment methods. Pain thresholds by algometry showed adequate statistical power (93.4%), confirming the robustness of findings, which was expected since sample size calculation was based on this primary endpoint. In contrast, CBPI scores showed lower power (42%), likely due to their subjective nature, dependent on caregiver perception. Nevertheless, the clinical data obtained through this tool were consistent with the observed improvements. These findings suggest that, to more sensitively assess perceived quality of life outcomes, a larger sample size would be required. Future studies with larger cohorts should be conducted to confirm the perceived effects of the intervention on pain and well-being.

In the present study, although both groups showed clinical improvement, dogs treated with the combined PBM and PEMF protocol demonstrated more pronounced and stable progression at the end of treatment. This result likely contributed to the high proportion of caregivers rating their dogs as “excellent” in terms of quality of life.

In addition to the analgesic effects of PBM and PEMF, it is important to consider that physiotherapy involves a higher level of interaction, physical manipulation, and cognitive stimulation, factors that may have positively influenced caregiver-reported well-being. Conversely, when bedinvetmab was used alone, although it promoted improvement, some caregivers subjectively reported a perception of fluctuating pain control, particularly near reapplication intervals. This perception was not captured by objective outcome measures and should therefore be interpreted cautiously. Previous studies have already indicated that dogs treated with PBM show increased physical activity, suggesting improved mobility and quality of life (Barale et al. 2022). Thus, while anti-NGF mAb monotherapy was effective, it may not be sufficient to ensure sustained clinical stability. PBM and PEMF, by enhancing pain relief while simultaneously promoting functional and emotional stimulation, represent essential resources in long-term multimodal therapeutic plans.

This study has some limitations, including a small sample size (*n* = 30) and a short follow-up period (90 days), which may restrict the generalizability of the findings. Although no adverse effects were observed, long-term monitoring is recommended to identify potential delayed reactions.

Although the study population was clinically characterized (age, sex, breed composition, body size, radiographic severity, and presence of OA in other joints), standardized measures of body condition score (BCS), muscle condition score (MCS), or validated sarcopenia/body composition assessments were not prospectively collected. As these variables may influence pain perception and functional impairment, their absence limits adjustment for potential confounding. Future studies should incorporate validated BCS/MCS scoring systems and objective measures of body composition or sarcopenia to strengthen domain clinical interpretability.

Another methodological limitation relates to the use of a single algometric measurement per time point, which deviates from the protocol recommended by Knazovicky et al. (2016), where repeated measurements and averaging are suggested to improve reliability. Although this approach was intentionally adopted to minimize animal discomfort, avoid nociceptive sensitization, and preserve the resting state required for assessment, it may have affected measurement accuracy and reproducibility. Previous studies indicate that consecutive mechanical stimuli, even at short intervals, can elicit anticipatory behavioral responses influenced by pain memory rather than by the applied pressure itself, particularly in dogs with chronic pain (Brown et al. 2007). Therefore, the algometric results should be interpreted considering this limitation.

A further limitation of the present study is the two-group design, which does not allow the individual effects of PBM and PEMF to be isolated or directly compared. A three-arm design (bedinvetmab only, bedinvetmab + PBM, and bedinvetmab + PEMF) would have enabled a clearer assessment of treatment-specific effects and potential synergism between modalities. However, this approach was not feasible due to ethical considerations, logistical constraints, and the sample size required to adequately power multiple comparisons. Consequently, the results should be interpreted as reflecting the effect of adding a combined PBM and PEMF physiotherapeutic protocol to bedinvetmab in dogs with chronic OA already undergoing multimodal pain management.

Another important limitation relates to the mechanistic interpretation of the combined use of PBM, PEMF, and the anti-NGF monoclonal antibody bedinvetmab. Although a targeted literature search was conducted, no studies were identified that directly evaluate the combined application of PBM and PEMF in dogs with osteoarthritis, nor their interaction with anti-NGF monoclonal antibody therapy. The mechanistic rationale discussed in this study is therefore based on biological plausibility and on independent evidence describing the effects of each modality separately. In particular, the reference by Enomoto et al. (2019) focuses on the role of nerve growth factor in osteoarthritis and the development of anti-NGF monoclonal antibodies, but does not address potential interactions with physiotherapeutic modalities. Consequently, any interpretation regarding complementary or synergistic mechanisms should be considered hypothesis-generating rather than confirmatory, and future mechanistic and controlled studies are warranted to clarify these interactions.

In addition, some dogs presented OA in joints other than the hip. Although this reflects the typical clinical scenario of polyarticular OA in dogs, it may have influenced overall pain perception and functional impairment. Nevertheless, all animals included had clinically and radiographically confirmed hip OA, and the therapeutic interventions and outcome assessments were specifically focused on the hip region. Future studies could benefit from the use of validated OA severity or multi-joint assessment scales to better quantify total disease burden and its impact on treatment response.

Moreover, the lack of formal blinding may have introduced observer bias, especially in subjective outcomes such as CBPI and QoL scores. Although the owners were not informed about the existence of different treatment groups or that other animals could be receiving additional therapies, the open-label design may still have introduced expectancy bias. Notably, the increased frequency of visits in the BPG, due to twice-weekly physiotherapy sessions, could have contributed to a greater perception of improvement by the owners. Therefore, the absence of full blinding should be considered when interpreting the superior clinical outcomes observed in the BPG.

Furthermore, for ethical reasons, the use of previously prescribed supportive medications (e.g., pregabalin, gabapentin, omega-3, and type II collagen) was maintained throughout the study. Discontinuing these interventions would compromise animal welfare and was therefore not feasible. While the inclusion of dogs receiving these treatments may represent a confounding factor, their use was stabilized for at least four weeks prior to enrollment and remained unchanged during the study. This ensured that any effects were already established and would not interfere with the evaluation of the primary treatments. Moreover, statistical analysis confirmed that the distribution of these medications was balanced between groups at baseline, minimizing potential bias. Including these cases increases the clinical relevance of the findings for dogs with chronic OA managed under multimodal analgesic protocols, in which adjunctive therapies are commonly maintained. The combination of bedinvetmab with PBM and PEMF was associated with improved analgesic outcomes and greater clinical stability in dogs with hip OA. Based on the exploratory nature of this study, these findings provide preliminary indication that physiotherapeutic modalities may serve as useful adjuvants to bedinvetmab within a multimodal approach to chronic pain management.

## Supplementary Information

Below is the link to the electronic supplementary material.

Supplementary Material 1

## Data Availability

The datasets generated during and/or analyzed during the current study are available from the corresponding author on reasonable request.
