# Supplementary material for: Bedinvetmab, alone or in combination with photobiomodulation and pulsed electromagnetic field therapy, on pain and quality of life in dogs with hip osteoarthritis
Source: Vet Res Commun. 2026 Mar 13;50(3):198. doi: 10.1007/s11259-026-11133-3 (PMC12987817; doi:10.1007/s11259-026-11133-3)
Supplement: Supplementary file 1 — Supplementary Material 1 [file 11259_2026_11133_MOESM1_ESM.docx]

Supplementary Table 1. Radiographic severity of hip dysplasia according to the FCI grading system in dogs treated with bedinvetmab alone (BG) ou bedinvetmab combined with physiotherapy (BPG).

| FCI grade | BPG (n=15) | BG (n=15) | p-value |
| --- | --- | --- | --- |
| C | 3 (20.0%) | 4 (26.7%) |  |
| D | 7 (46.7%) | 6 (40.0%) |  |
| E | 5 (33.3%) | 4 (26.7%) | 0.894 |

Data are presented as number of dogs (%). Groups comparisinon was performed using chi-square test.
